# Critical role of Sirtuin 6 (SIRT6) in osteoarthritis chondrocytes: the interaction among various biological processes

**DOI:** 10.1080/07853890.2026.2638000

**Published:** 2026-03-05

**Authors:** Ning Ding, Zongru He, Qingshan Yang

**Affiliations:** Sports Medicine Ward, Gansu Provincial Hospital, Lanzhou, China

**Keywords:** Osteoarthritis, Sirtuin 6, biological processes, signal pathway

## Abstract

**Background:**

OA (osteoarthritis) is a joint disease that causes drastic economic and financial burdens on people worldwide. Cartilage destruction and synovial inflammation contribute to joint disability, pain, weakness and deformity. Therefore, treatment of OA based on molecular regulation is important. SIRT6 (Sirtuin 6) possesses deacetylase activity and is responsible for many ageing-related biological processes.

**Purpose:**

SIRT6 activity is affected by age, inflammation, and oxidative stress. However, there is a need to elucidate its role in OA. Chondrocytes are one of the most essential components of articular cartilage. Increasing evidence indicates that SIRT6 plays a critical role in regulating chondrocyte functions in OA. This review aimed to reveal the mechanisms by which SIRT6 regulates OA in chondrocytes.

**Discussion:**

SIRT6 is also involved in cartilage inflammation, chondrocyte senescence, redox balance, and DNA damage repair. Various biological processes regulate OA development and progression. SIRT6 regulation is an elaborate network that cannot be dissociated. Although an increasing number of studies have focused on SIRT6 function, more detailed research papers are lacking. Therefore, the interwoven functions of SIRT6 should be investigated in future studies.

**Conclusion:**

This review describes the multi-faceted protective roles of SIRT6 in OA chondrocytes. SIRT6 participates in many biological processes and acts as a key node interacting with many cytokines to regulate OA progression. With further researches, the mechanisms of it will be more explicit and it might be a potential therapeutic target for OA treatment.

## GRAPHICAL ABSTRACT

The overview of SIRT6 functions in OA chondrocyte.

**Figure:**
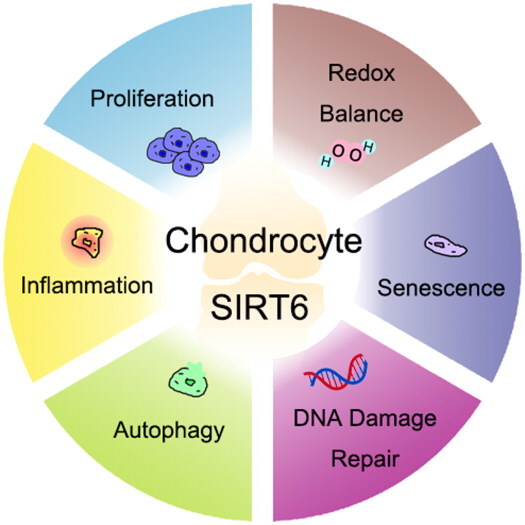

## Introduction

Osteoarthritis (OA), the most common type of arthritis, is mainly characterised by progressive articular cartilage erosion and secondary synovitis [1], resulting in joint pain and movement disorders. Although it is the focus of studies worldwide, finite therapies can be applied in the current treatment of OA. Surgery remains the primary final treatment for OA, especially for knee and hip osteoarthritis. Chondrocytes, the main cellular components of the articular cartilage, are predominantly involved in physiological and pathological processes. In the OA microenvironment, chondrocytes regulate the progression of OA.

In mammals, SIRT6 (Sirtuin 6) is one of 7 members in the SIRT family. Different sirtuin function in varying subcellular compartments, including the nucleus, cytoplasm and mitochondria. Among the sirtuin family, SIRT6 is associated with life span [2,3]. SIRT6 has emerged as a therapeutic target for age-related diseases in many studies. SIRT6 is involved in cellular differentiation, senescence, and inflammation. Previous studies show that it can regulate OA [4], rheumatoid arthritis (RA) [5], osteoporosis (OP) [6], and other diseases. In this review, we focused on the function of SIRT6 in OA chondrocyte. Multiple SIRT6-related biological processes have influence on OA progression. It has been reported that SIRT6 level in OA cartilage is lower than that in normal cartilage, with cartilage thickness decreasing with aging [7]. Therefore, SIRT6 is critical for OA regulation in cartilages and may be a novel target for future OA treatment. Accordingly, this review describes the protective functions of SIRT6 in OA chondrocytes.

## Fundamental feature of SIRT6

SIRT6 is a NAD^+^-dependent class III histone deacetylase in the nucleus. It deacetylates histone 3 at lysine9, 13, 18 and 56 [8–11]. Various biological processes rely on it including DNA damage repair [12,13], cellular senescence [14,15] and inflammatory responses [5,16]. It has been reported that SIRT6 regulates postnatal growth plate proliferation [17] and inhibits bone loss [4], and is vital for regulating bone metabolism.

## Redox balance

Maintaining redox balance is critical for cells. Oxidative stress is a direct risk factor for cellular injury in patients with OA. Accumulating evidence suggests that SIRT6 regulates redox balance and protects against oxidative stress [12,18]. When chondrocytes are exposed to menadione and DMNQ, which promote H_2_O_2_ generation, sulfenylation of SIRT6 is upregulated and SIRT6 is oxidised. SIRT6 suppresses deacetylation in the presence of H_2_O_2_. Meanwhile, Prxs (peroxiredoxins) undergo hyperoxidation, thereby inhibiting their peroxidase activity and increasing oxidative stress. In contrast, when SIRT6 is overexpressed, peroxiredoxin 1 (Prx1) and sulfiredoxin (Srx), as antioxidant cytokines, are up-regulated. Srx restores Prx activation status by reducing hyperoxidation [19]. In chondrocytes, SIRT6 regulates redox balance by increasing Prx1 and Srx antioxidant levels and decreasing the level of thioredoxin interacting protein (TXNIP), a thioredoxin inhibitor. Therefore, SIRT6 promotes Prx and Srx expression to restrict oxidative stress and facilitate cellular antioxidant capabilities [20]. It has been reported that TXNIP is involved in regulating redox balance, which can enhance oxidative stress [21]. Moreover, SIRT6 can directly bind to Nrf2 [18], decrease the level of TXNIP, and reduce reactive oxygen species(ROS) generation [22]; TXNIP can be inhibited by Nrf2 [23], indicating that the SIRT6-Nrf2-TXNIP axis regulates chondrocyte redox balance. Apparently, Prx/Srx and Nrf2 can be regulated by SIRT6 to affect redox balance.

## Cellular senescence

**Figure 1.**
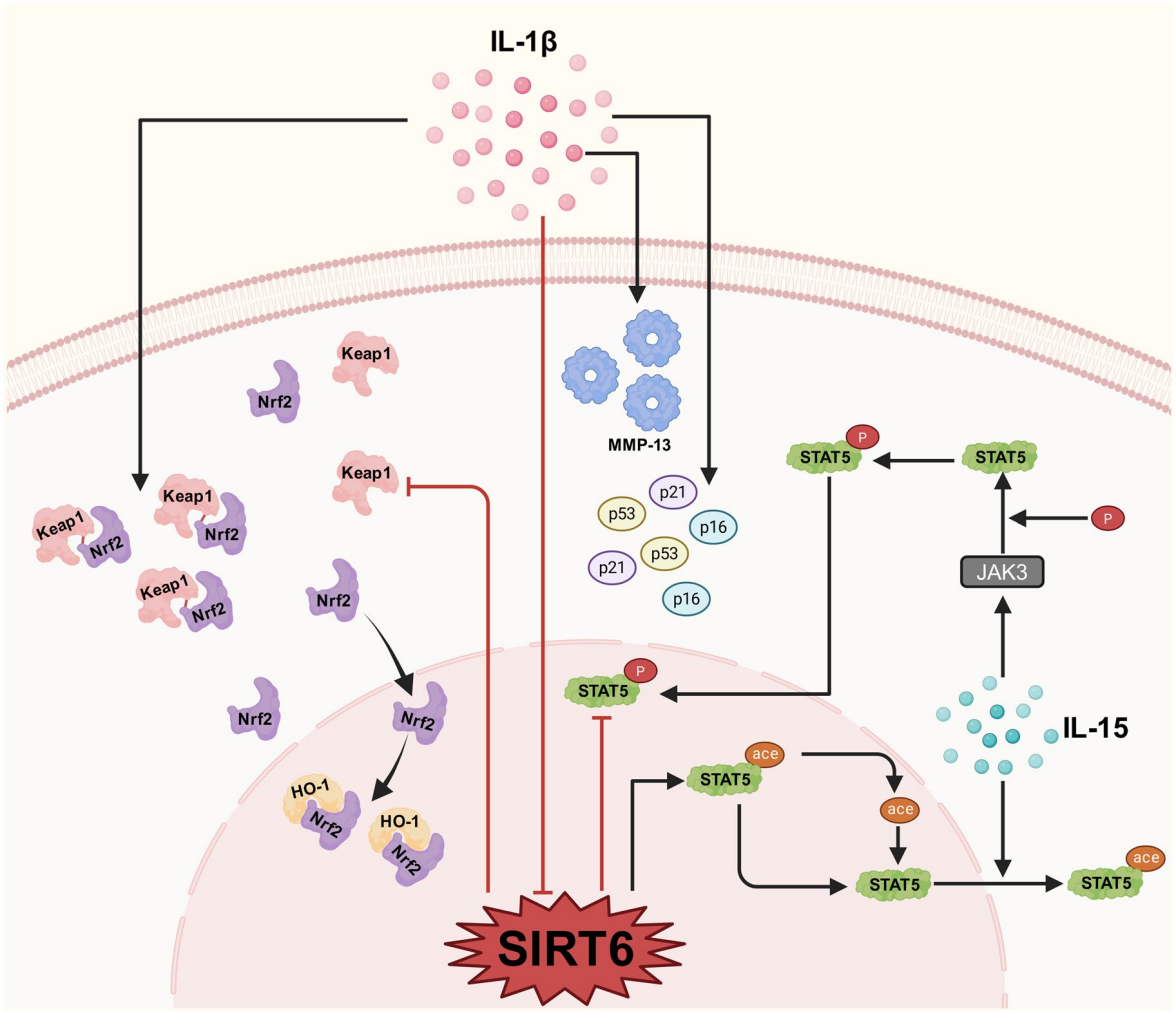
SIRT6 inhibits chondrocyte senescence.

Chondrocyte senescence, resulting from DNA damage and telomere injury, directly contributes to OA [24,25] (Figure 1). Low SIRT6 levels are observed in senescent chondrocytes. IL-1β increases the secretion of MMP-13 [7], p53, p21 and p16, while suppressing SIRT6 expression [26] and subsequently promoting chondrocyte senescence. In IL-1β-induced senescence, Keap1 binds to Nrf2 and forms cytoplasmic complex thereby negatively regulating Nrf2 function. Once SIRT6 is overexpressed, Keap1 is down-regulated, and the Keap1/Nrf2 complex is disrupted. Nrf2 then translocates to the nucleus and combines with HO-1. Apparently, SIRT6 inhibits chondrocyte senescence *via* the Keap1/Nrf2/HO-1 signalling pathway [26]. Notably, overexpressed SIRT6 reverses IL-1β-inducing the expression of MMP-13 and inhibits the expression of collagen II [7]. Moreover, IL-15 upregulates STAT5 acetylation, which can be deacetylated at lysine 163 (K163) by SIRT6. In senescent chondrocytes, acetylation and phosphorylation of STAT5 are upregulated. STAT5 is a direct substrate of SIRT6. SIRT6 overexpression suppresses STAT5 phosphorylation by IL-15/JAK3 and inhibits STAT5 translocation into the nucleus. The IL-15/JAK3/STAT5 axis is crucial for promoting cellular senescence and is repressed by SIRT6 [27].

## DNA damage repair

**Figure 2.**
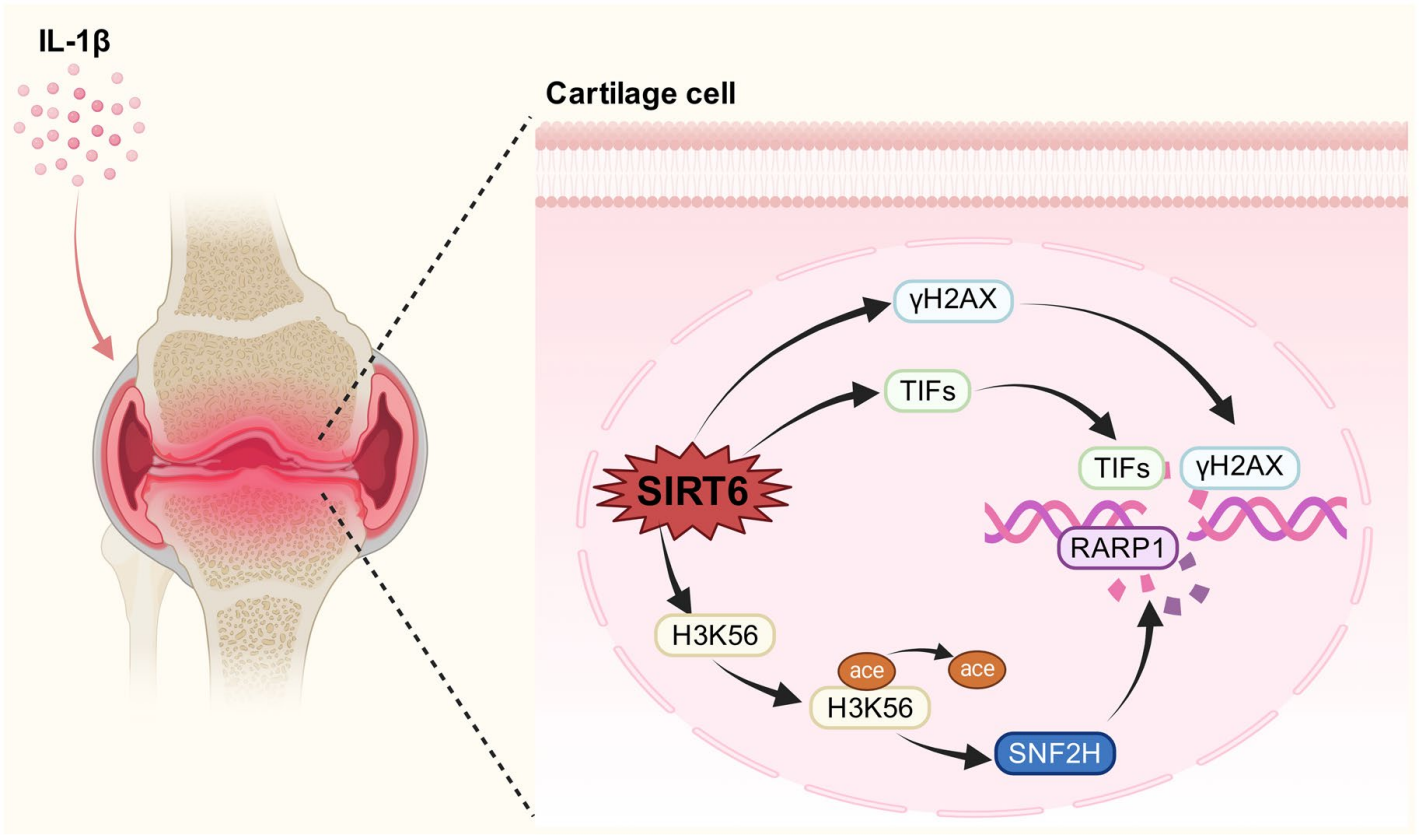
SIRT6 takes part in DNA damage repair.

With increasing age, the ability to repair DNA damage is attenuated. SIRT6 is weakly expressed in OA cartilage (Figure 2). SIRT6 activation by MDL-800 decreases DNA damage and promotes DNA damage repair in OA chondrocytes [28]. During IL-1β-induced OA chondrocyte, SIRT6 is inhibited and DNA damage is enhanced. Meanwhile, γH2AX and TIFs (telomere dysfunction-induced foci) increase and accumulate at DNA damage sites and abnormal telomeres [25]. Since γH2AX has function of assemble in DNA double-strand breaks, γH2AX is regarded as an early sign of DNA damage [29]. Apart from γH2AX and TIFs, Chk1 (Checkpoint kinase 1) can also be activated by DNA damage [26]. Reportedly, Chk1 is a cell cycle related kinase can be activated in terms of DNA damage [30] which is another key marker of DNA damage. Overexpression of SIRT6 reverses DNA damage induced by IL-1β in chondrocyte to promote DNA damage repair [26]. Moreover, SIRT6 takes part in deacetylating H3K56 to propel SNF2H [31] and makes PARP1 (poly ADP-ribose polymerase 1) [32] recruit at DNA double-strand break sites to repair them. To sum up, when DNA damages, SIRT6 makes many DNA repairing cytokines recruiting damage sites to repair them and remodels chromatin.

## Chondrocyte autophagy

**Figure 3.**
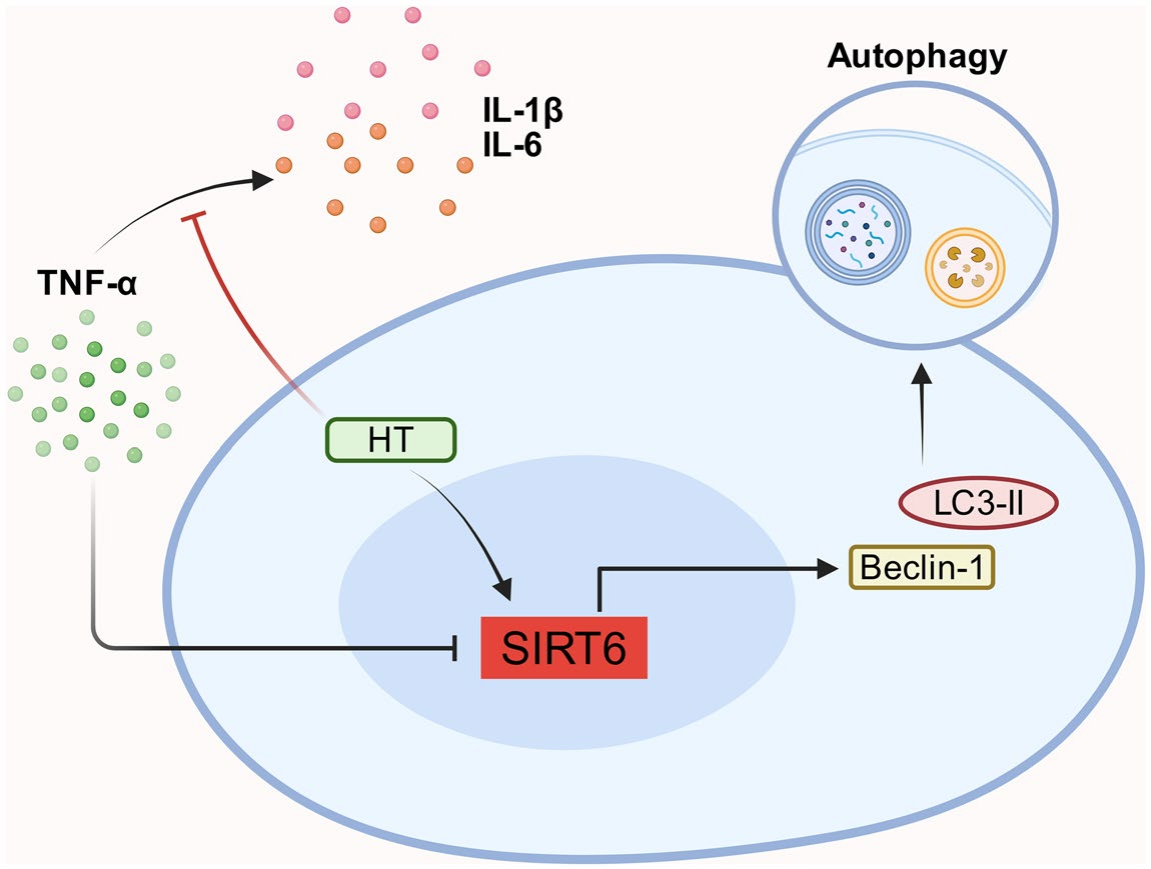
SIRT6 promotes cellular autophagy.

Autophagy is a self-protective mechanism that provides cellular nutrients to resolve abnormal cells and maintain cellular homeostasis [33]. This is a critical factor that causes inflammation [34] (Figure 3). TNF-α exerts an influence on chondrocyte, resulting in the upregulation of IL-1β and IL-6, a process that can be mitigated by Hydroxytyrosol (HT). HT increases the levels of SIRT6, LC3-II and Beclin1 even under TNF-α-induced inflammation. SIRT6 silencing increases MCP-1 levels, but decreases Beclin-1 and LC3-II levels [35]. SIRT6 mediates HT-inducing chondrocyte autophagy.

## Inflammation

**Figure 4.**
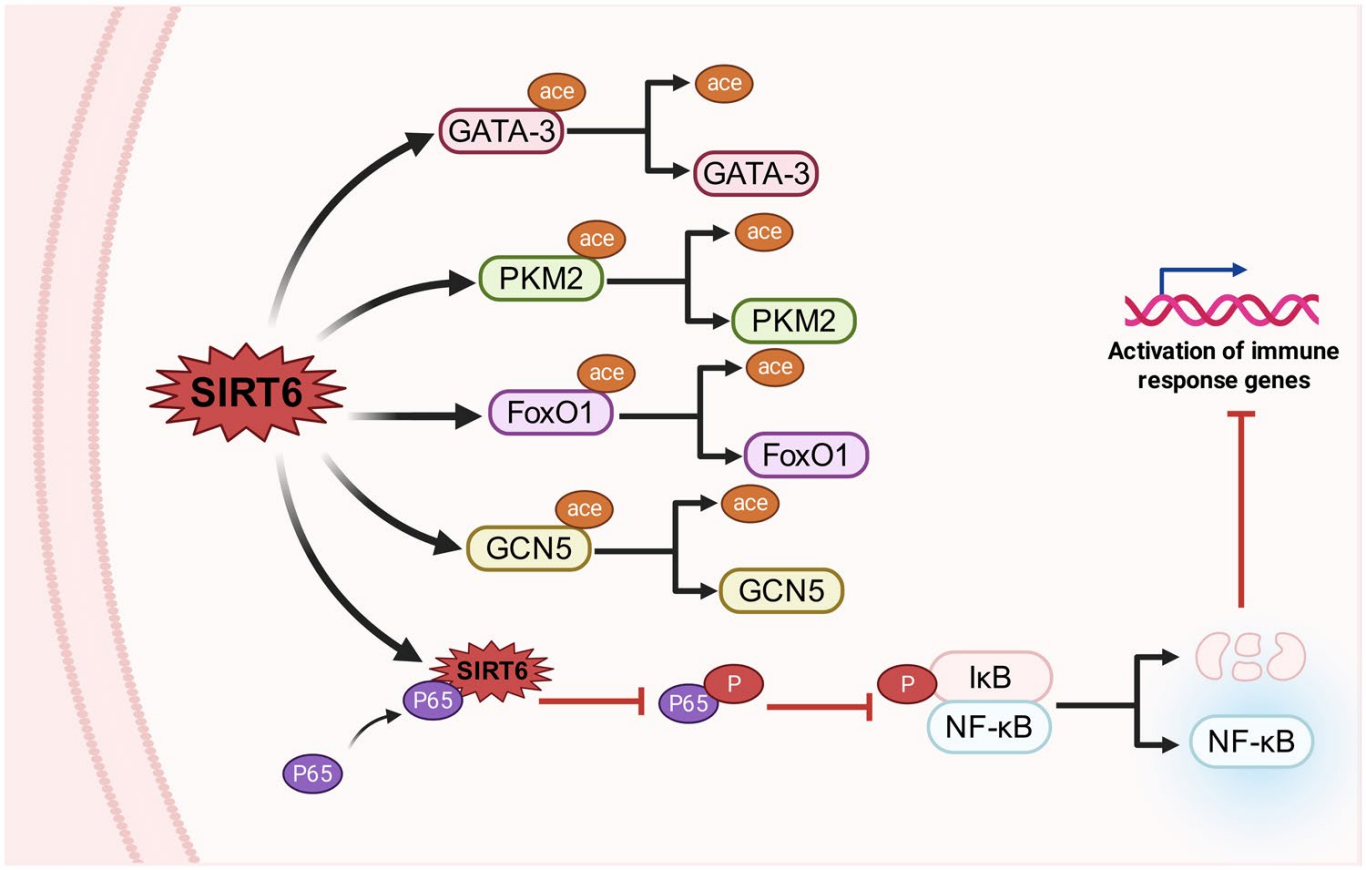
SIRT6 suppresses inflammation progression.

In OA chondrocytes, COX-2 and iNOS, as inflammation mediators, are induced by IL-1β [36]. For IL-1β-inducing OA in chondrocytes, p65 is phosphorylated and translocated into the nucleus to activate downstream targets [37,38] and SIRT6 is inhibited. SIRT6 can bind to p65 of NF-κB to inhibit its phosphorylation through the binding site of SIRT6 (amino acids 34–274 domain) [39] and subsequently repress NF-κB-involved in inflammation [40]. Phosphorylation of p65 is suppressed, phosphorylated p65 entering the nucleus is blocked, and deacetylation of H3K9 and H3K56 of NF-κB target genes from SIRT6 restrains chondrocyte inflammation [7,20,41]. In many other cells, SIRT6 inhibits inflammation by deacetylating GCN5, FoxO1 [42], PKM2 [43] and GATA-3 [44] (Figure 4). Inflammation is a dominant pathological progression in OA. SIRT6 inhibits inflammation mainly through downstream targets of NF-κB pathway.

## Chondrocyte proliferation

**Figure 5.**
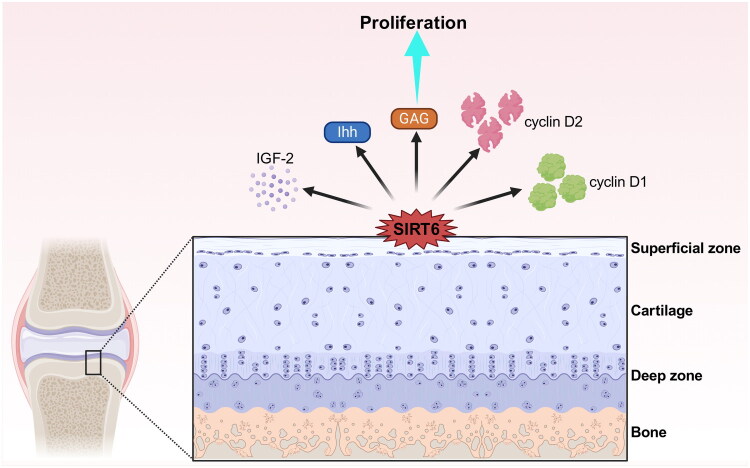
SIRT6 has a protective effect on chondrocyte proliferation.

SIRT6 is conducive to chondrocyte proliferation [25] (Figure 5). SIRT6 is predominantly expressed in the superficial cartilage. In SIRT6-depletion mice, proliferation and hypertrophic zones of the growth plate are attenuated [25]. SIRT6 inhibition suppresses the expression of cyclin D1 and D2 as well as the Indian hedgehog (Ihh) pathway in chondrocyte [17]. Ihh mediates SIRT6 regulation of chondrocyte proliferation and differentiation [24]. Glycosaminoglycan (GAG), an important component of the ECM, is up-regulated by SIRT6 in the cartilage [45], demonstrating the protective function of SIRT6 in ECM preservation. IGF-1 regulates the ECM of cartilage by activating and phosphorylating AKT [46–48]. SIRT6-deficiency inhibits ICF-1 expression [49]. Notably, in OA cartilages, SIRT6 is upexpressed in chondrocytes, which form clusters and proliferate inside them [50]; this is regarded as a self-repair mechanism [51]. At the same time, ECM generation induced by SIRT6 provides stable microenvironments.

## Discussion and perspective

SIRT family contains 7 members. This review shows SIRT6 takes part in regulating redox balance, inhibiting chondrocyte senescence, promoting DNA damage repair, inducing autophagy, repressing cellular inflammation and facilitating proliferation. Apart from it, other SIRTs also have influence on chondrocytes. SIRT1 participates in regulating chondrocyte inflammation [52] and conserving cartilage ECM induced by Nrf-2/HO-1 pathway [53]. SIRT2 alleviates chondrocyte inflammation through PCK1 [54] and promotes cartilage repair by NOD-like receptor protein 3 (NLRP3)/NF-κB [55]. SIRT3, SIRT4, SIRT5 and SIRT7 are less focused, but it has been confirmed that they have protective functions to chondrocytes and cartilage in OA [56–58]. Interestingly, the sympathetic nervous system (SNS) is confirmed to regulate chondrocyte releasing exosomes [59]. Chondrocyte apoptosis and senescence can be inhibited by NE through activating β1-adrenergic receptor [59,60]. NE activating β1-adrenergic receptor suppresses SIRT6 expression, which promotes chondrocyte inflammation [59]. The brain-sympathetic-bone axis emerges, which proposes a new OA regulation mechanism [61].

There exist limitations on the mechanisms of SIRT6 in OA chondrocytes. Several studies revealed that some cytokines are regulated by SIRT6 and other members. It is unknown whether there are synergism or interactions among them. Moreover, current studies can hardly reveal comprehensive and integrated mechanisms of SIRT6 on OA chondrocytes.

## Conclusions

This review summarises the accumulating data that reveal the favourable functions of SIRT6 in chondrocytes and its ability to protect cartilage from OA. SIRT6 plays a critical role in various biological processes in OA, including inflammation, senescence, autophagy, and metabolism. Many cytokines are associated with SIRT6 across different signalling pathways involved in OA regulation. These findings encourage further studies on SIRT6 in the context of OA pathogenesis and development.

## Data Availability

Data sharing is not applicable to this article as no new data were created or analyzed in this study.

## Open scholarship

This article has earned the Center for Open Science badges for Open Data and Open Materials. The data and materials are openly accessible at https://www.ncbi.nlm.nih.gov/ and https://www.ncbi.nlm.nih.gov/.
